# Iron Hack - A symposium/hackathon focused on porphyrias, Friedreich’s ataxia, and other rare iron-related diseases

**DOI:** 10.12688/f1000research.19140.1

**Published:** 2019-07-19

**Authors:** Gloria C. Ferreira, Jenna Oberstaller, Renée Fonseca, Thomas E. Keller, Swamy Rakesh Adapa, Justin Gibbons, Chengqi Wang, Xiaoming Liu, Chang Li, Minh Pham, Guy W. Dayhoff II, Linh M. Duong, Luis Tañón Reyes, Luciano Enrique Laratelli, Douglas Franz, Segun Fatumo, ATM Golam Bari, Audrey Freischel, Lindsey Fiedler, Omkar Dokur, Krishna Sharma, Deborah Cragun, Ben Busby, Rays H.Y. Jiang

**Affiliations:** 1 Department of Molecular Medicine, Morsani College of Medicine, University of South Florida, MDC 7, Tampa, FL, 33612, USA; 2 Global and Planetary Health, College of Public Health, University of South Florida, USF Genomics Program, 3720 Spectrum Blvd, Tampa, FL, 33612, USA; 3 Morsani College of Medicine, University of South Florida, 12901 Bruce B Downs Blvd, Tampa, FL, 33612, USA; 4 University of South Florida, USF Genomics Program, 3720 Spectrum Blvd, Tampa, FL, 33612, USA; 5 Center for Urban Transportation Research, University of South Florida, 4202 E. Fowler Avenue, CUT100, Tampa, FL, 33620, USA; 6 Department of Chemistry, University of South Florida, 4202 E. Fowler Avenue, CHE 205, Tampa, FL, 33620-5250, USA; 7 College of Public Health, University of South Florida, 13201 Bruce B. Downs Blvd., MDC 56, Tampa, FL, 33612, USA; 8 Moffitt Cancer Center, Tampa, FL, 33612, USA; 9 Department of Cell Biology, Microbiology and Molecular Biology, University of South Florida, 4202 East Fowler Ave, ISA 2015 Tampa, FL, 33620, USA; 10 MRC/UVRI and LSHTM (Uganda Research Unit), Entebbe, Uganda; 11 Department of Computer Science and Engineering, University of South Florida, Tampa, FL, USA; 12 University of South Florida, Tampa, FL, 33620, USA; 13 National Library of Medicine, 8600 Rockville Pike, Bethesda, MD, 20894-6075, USA

**Keywords:** Hackathon, Data Science, Ataxia, Porphyria, Rare Diseases, Friedreich’s Ataxia, Clinical Informatics, Bioinformatics

## Abstract

**Background**: Basic and clinical scientific research at the University of South Florida (USF) have intersected to support a multi-faceted approach around a common focus on rare iron-related diseases. We proposed a modified version of the National Center for Biotechnology Information’s (NCBI) Hackathon-model to take full advantage of local expertise in building “Iron Hack”, a rare disease-focused hackathon. As the collaborative, problem-solving nature of hackathons tends to attract participants of highly-diverse backgrounds, organizers facilitated a symposium on rare iron-related diseases, specifically porphyrias and Friedreich’s ataxia, pitched at general audiences.

**Methods**: The hackathon was structured to begin each day with presentations by expert clinicians, genetic counselors, researchers focused on molecular and cellular biology, public health/global health, genetics/genomics, computational biology, bioinformatics, biomolecular science, bioengineering, and computer science, as well as guest speakers from the American Porphyria Foundation (APF) and Friedreich’s Ataxia Research Alliance (FARA) to inform participants as to the human impact of these diseases.

**Results**: As a result of this hackathon, we developed resources that are relevant not only to these specific disease-models, but also to other rare diseases and general bioinformatics problems. Within two and a half days, “Iron Hack” participants successfully built collaborative projects to visualize data, build databases, improve rare disease diagnosis, and study rare-disease inheritance.

**Conclusions**: The purpose of this manuscript is to demonstrate the utility of a hackathon model to generate prototypes of generalizable tools for a given disease and train clinicians and data scientists to interact more effectively.

## Introduction

### Iron Hack: Genesis of a new hackathon model

Hackathons are an effective avenue for the generation of software prototypes in the biomedical informatics space, several of which have been sponsored by the National Institutes of Health (NIH NCBI). A long-standing interest and active research programs on rare diseases, including Friedreich’s ataxia and porphyrias at the University of South Florida (USF), prompted us to modify the traditional NCBI Hackathon model and initiate a specific disease-focused hackathon ^1– 3^. Our event “Iron Hack” was named after the rare diseases upon which we focused.

Because of the diverse scientific and bioinformatic backgrounds of hackathon participants, the organizers felt it necessary to have a symposium on rare iron-related diseases, and specifically ataxia and porphyrias, in the early part of each of the three days. During the symposium, renowned scientists, clinicians and investigators in rare iron-related disease research covered the major aspects of Friedreich’s ataxia, porphyrias and sideroblastic anemia and emphasized pressing questions that need to be addressed for advancement of the field. As a result of the hackathon, we developed resources that are relevant not only to rare iron-related diseases, but also to other rare diseases and some general bioinformatics problems. The objective of this report is to demonstrate the utility of a hackathon model to develop generalizable tools for evaluation, diagnosis, and management of a given disease.

### Rare iron-related diseases: Hurdles to overcome

Rare diseases have a large impact on the population, with 7,000 orphan diseases collectively affecting about 1 in 10 to 20 people ^4^. These diseases place a heavy burden on patients and families, with a diagnosis taking up to ten years to identify, if ever. Limited patient-numbers and resources for each disease severely hamper research, prognoses, diagnoses, and treatments. Although quite disparate in symptoms, these conditions all stem from problems with iron metabolism or heme synthesis. A brief description of these rare diseases is provided below to provide context for the “Iron Hack” team projects.

### Porphyrias

***Biochemical basis and clinical manifestation.*** Porphyrias are a group of rare metabolic disorders caused by malfunction of the enzymes involved in heme biosynthesis ^2, 3, 5^. There are eight different types of porphyria, each of which arises from mutation(s) in the genes for each of the eight enzymes of the heme biosynthetic pathway ^2, 3, 6, 7^. With the exception of porphyria cutanea tarda (PCT), all other seven porphyrias are inherited as autosomal-dominant, autosomal-recessive, or X-linked traits ^2, 6, 8, 9^.

All porphyrias are characterized by the accumulation and excretion of porphyrins or porphyrin precursors, though each type has disparate clinical manifestations (including neurovisceral and/or cutaneous symptoms) depending upon which enzyme of the pathway is defective ^10^. In general, neurological disturbances are manifested in the form of acute attacks (e.g., extreme abdominal and chest pain, vomiting, confusion, constipation, fever, high blood pressure, low blood sodium levels, and seizures), while photosensitivity is at the root of cutaneous manifestations (e.g., skin blistering, redness, scarring, and pain when exposed to the sun) ^3, 9, 11, 12^. Suggested treatments and disease-outcomes also vary with the particular pathway-defect ^10, 13– 19^.

***Diagnosis.*** Acute porphyrias can prevail undiagnosed for 10–15 years following the onset of symptoms ^19^. Perhaps not surprisingly, diagnosis of porphyrias remains challenging: they are rare, and their symptoms are nonspecific, often mimicking other, more common disorders ^11^. Thus once porphyria clinical symptoms are recognized, biochemical laboratory testing should be performed to identify the specific type of porphyria ^10^. Genetic testing, normally targeted gene sequencing, becomes critical to define the mutation(s) in a family and make genetic counseling possible. However, diagnosis of porphyrias are often overlooked due to, a large extent, the difficulty in performing the specific biochemical assays and absence of specialized porphyria centers, particularly in developing countries ^20– 22^.

***Prevalence.*** In the United States, porphyrias collectively afflict fewer than 200,000 people, with similar prevalence in the European Union ^4, 10, 23^. Estimates of porphyria prevalence vary by type, with values of 1 in 10,000 for the most common type of porphyria, PCT ( OMIM 176090) and 1 in 1,000,000 for congenital erythropoietic porphyria (CEP; OMIM 263700) ^10^. Many people with a genetic mutation associated with the disease never experience signs or symptoms, a phenomenon known as incomplete or reduced penetrance ^24^.

### Friedreich’s ataxia

***Biochemical basis and clinical manifestation.*** Friedreich’s ataxia (FRDA, FA; OMIM 229300) is a rare autosomal-recessive disease associated with progressive spinocerebellar ataxia, cardiomyopathy, scoliosis, diabetes, and vision and hearing impairment ^1, 25^. Most FRDA patients are largely asymptomatic during the first 5 – 10 years of life. But, with advancing gait and limb ataxia, they require use of a wheelchair and are unable to perform daily activities independently, often during adolescence ^25, 26^.

Symptoms result from reduced synthesis of the mitochondrial protein frataxin, an iron chaperone that, by shielding this metal, prevents the production of reactive oxygen species (ROS) and renders it bioavailable as ferrous iron ^26– 29^. When frataxin levels are low, iron accumulates in the mitochondria, largely in an oxidized and insoluble form ^30, 31^. The accumulated iron can participate in Fenton chemistry leading to formation of extensive reactive oxygen species (ROS) that cause damage and cell death. While there is a general agreement that frataxin is critical for mitochondrial iron metabolism and cellular iron homeostasis, its precise biological role remains a controversial matter ^26, 32, 33^. Involvement of frataxin in 1) iron delivery to the iron–sulfur cluster assembly and repair machinery, 2) repair of oxidatively inactivated [3Fe–4S] aconitase to yield an active enzyme, 3) delivery of ferrous iron to ferrochelatase for heme biosynthesis, 4) detoxification of iron by catalyzing the oxidation of Fe(II) to Fe(III) and storing the metal as a ferrihydrite mineral within structurally organized frataxin oligomers are among the reported functions ascribed to frataxin ^26, 31, 34– 41^. Despite the lack of consensus, frataxin and heme biosynthesis are linked. Frataxin may participate in the assembly of the [2Fe-2S] cluster, an essential cofactor for an active human ferrochelatase, the terminal enzyme of the heme biosynthetic pathway ^42– 44^. Alternatively, by maintaining and chaperoning iron in a reduced form, frataxin may donate Fe(II) to ferrochelatase, which has strict physiological specificity for Fe(II) as substrate ^37, 39, 44– 46^. Clearly, a combination of these two functional possibilities cannot be ruled out.

***Prognoses and treatment.*** Presently, there is neither a cure nor a U.S. Food and Drug Administration (FDA)-approved treatment for FRDA ^47^. Advances in understanding the underlying mechanism of FRDA, in particular the recognition that frataxin deficiency is the root cause of FRDA, have prompted the development of therapeutic strategies. Since increased oxidative stress and mitochondrial respiratory chain dysfunction have been associated with the pathogenesis of FRDA, antioxidants and inhibitors of free radical formation (e.g., idebenone, L-acetylcarnitine, resveratrol, and RT001 and other deuterated polyunsaturated fatty acids) have been assessed as a promising treatment option ^47– 50^. Iron chelators, such as deferiprone, have been considered as a therapeutic approach of FRDA by controlling iron accumulation and decreased frataxin synthesis ^29, 51^. Regulation of frataxin gene expression by increasing either histone acetylation or transcription of the frataxin gene, represents yet another treatment possibility being explored ^52, 53^. Histone deacetylase inhibitors reverse or, at least, diminish silencing and the reduced transcription of the frataxin gene observed in FRDA patients ^54^. While protein interferon- increases transcription of the frataxin gene and consequent production of the frataxin, its therapeutic benefit remains to be established ^52, 55^. Frataxin gene replacement is also being developed for as a potential treatment for FRDA. Because of the scope of this report, an evaluation of the therapies for FRDA can be neither extensive nor complete ^47^.

***Prevalence.*** FRDA affects about 1 in 150,000 individuals of Caucasian descent and accounts for 50% of overall cases of hereditary ataxia and for 75% of those with onset before age 25 ^1, 25, 56, 57^.

### Genetics of porphyrias and FRDA: a tractable problem?

The robust evidence suggesting these devastating, rare diseases named porphyrias can largely be pinpointed to dysfunction in a single pathway of eight enzymes, caused by mutation(s) inherited in well-understood, classical Mendelian patterns made them an attractive case for potentially-impactful tool-development. However, even in these seemingly straightforward cases of diseases exhibiting classical Mendelian inheritance, disease phenotypes are not entirely explained by the presence of known pathogenic variants. The discrepancy between the low penetrance of symptomatic patients for autosomal-dominant acute intermittent porphyria (AIP; OMIM 176000) and the high frequency of pathogenic mutations led Chen *et al*. to propose that predisposing- or protective-modifier genes alter expression of the AIP phenotype ^58^. Indeed, a small number of modifier-genes, regulatory and pathophysiological mechanisms have since been identified to contribute to onset of porphyrias, though these findings remain insufficient to explain the disease-penetrance puzzle ^8, 20^.

In rare diseases resulting from trinucleotide copy-number repeat-variation, such as FRDA, there is some degree of relationship between severity of disease phenotype and copy-number ^57, 59^. In FRDA, expanded trinucleotide (GAA) tracts in intron 1 of the FXN gene, commonly between 600 and 900 repeats, result in pathologically decreased levels of frataxin ^60– 62^. However, number of trinucleotide repeats are not reliably predictive of disease severity, further suggesting the importance of as-yet unknown modifying genes or environmental factors that may contribute to disease outcomes ^47^.

The number of disease-phenotypes entirely decided by single-gene variants are in the minority ^63^. Most inherited diseases are likely to have a more complicated etiology determined by some combination of genomic variants, impacted by myriad environmental factors as well.

### Critical gaps Iron-Hack projects sought to address

We organized Iron Hack to address these challenges, including the great need for genomics tools to handle rare-disease data, such that new data-mining concepts and computational tools could be developed and further adapted to serve the rare-disease communities. We established five Iron Hack teams to develop five computational-tool prototypes broadly focused on (1) *exploration of consumer-genomics data*, (2) *large-scale RNAseq data mining*, (3) *genomic data visualization*, (4) *rare-disease variants discovery*, and (5) *genotype-to-phenotype mapping*. These team-efforts have led to the convergence of iron-research communities and genomics data-science researchers to produce promising computational tools, strengthened through an iterative process of soliciting ideas and feedback from domain experts.

The remainder of this report is organized into subsections by project, beginning with a detailed description for the five projects, the motivations behind them and the gaps they seek to fill. We next describe the methodologies and implementations of the projects into usable software applications, how to operate the software applications, and results produced using the software applications. Finally, we discuss the pros and cons of this new highly-interdisciplinary and community-driven twist on more traditional hackathons.

## Project descriptions and goals

### Project 1: UPWARD

**Figure 1.**
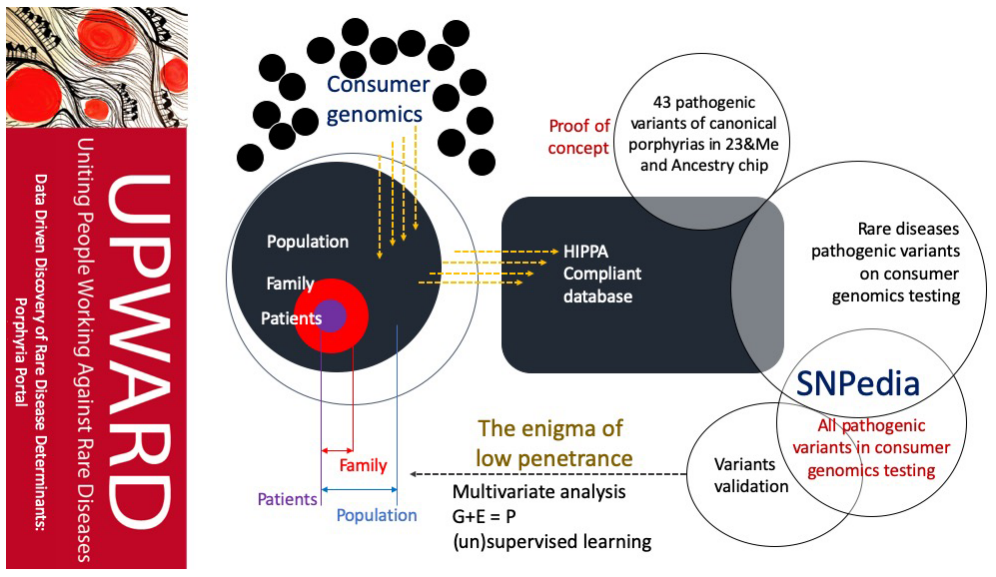
UPWARD - Uniting People Working Against Rare Disease. UPWARD opens with a web interface designed to clearly communicate research and advocacy goals to the public, request consent and gather data in a HIPPA-compliant manner.

Uniting People Working Against Rare Diseases (UPWARD) will be a Health Insurance Portability and Accountability Act (HIPAA)-compliant database which will allow people with rare diseases to declare interest in participating in research studies, and subsequently share their personal disease stories, clinical symptoms, and consumer genetic testing data with researchers and clinicians. Figure 1 shows that, as consumer-genetic testing data are submitted, they are analyzed alongside 43 porphyria-related pathogenic SNPs currently held in UPWARD. A set of statistical computation and machine learning methods can be used downstream to parse out the novel modifiers of diseases as well as the interactions of genetic loci underlying pathologies. This information with be compiled and analyzed within UPWARD using, in part, a program which identifies all rare disease-related pathogenic or likely-pathogenic Single Nucleotide Polymorphisms (SNPs) that are currently included on SNP microarray chips used by common consumer genetic testing companies. Table 1 shows that variants sourced from ClinVar, a crowdsourced genotype-phenotype database hosted by NCBI, against consumer-genetics data sourced from Illumina OmniExpress and GSA microarray chips used by Ancestry and 23andMe. These resulting 43 variants will be used in analysis of patient-submitted consumer-genomics results. The goal of this platform is to facilitate data-driven discovery of rare-disease determinants, such as modifiers that affect penetrance, by leveraging the growing data of consumer genomics. To facilitate use of this database, UPWARD has focused its tools to benefit people living with porphyria, and porphyria research as a whole.

**Table 1.** List of porphyria-related pathogenic SNPs. UPWARD includes a tool built to map highly-pathogenic and likely-pathogenic porphyria-associated variants.

Name	Gene	RSID	Chip
NM_000374.4(UROD):c.603A>G (p.Pro201=)	UROD	rs2228084	GSA
NM_000374.4(UROD):c.842G>A (p.Gly281Glu)	UROD	rs121918057	GSA
NM_000374.4(UROD):c.842G>T (p.Gly281Val)	UROD	rs121918057	GSA
NM_000374.4(UROD):c.874C>G (p.Arg292Gly)	UROD	rs121918059	GSA
NM_000374.4(UROD):c.912C>A (p.Asn304Lys)	UROD	rs121918065	GSA
NM_000374.4(UROD):c.932A>G (p.Tyr311Cys)	UROD	rs121918061	GSA
NM_000374.4(UROD):c.995G>A (p.Arg332His)	UROD	rs121918066	GSA
NM_000309.4(PPOX):c.-90G>T	PPOX	rs115158839	GSA
NM_001122764.1(PPOX):c.199delC (p.Leu67Terfs)	PPOX	rs786204784	GSA
NM_001122764.3(PPOX):c.502C>T (p.Arg168Cys)	PPOX	rs121918325	GSA
NM_000097.5(CPOX):c.814A>C (p.Asn272His)	CPOX	rs1131857	GSA
NM_000410.3(HFE):c.187C>G (p.His63Asp)	HFE|LOC108783645	rs1799945	GSA
NM_000410.3(HFE):c.193A>T (p.Ser65Cys)	HFE|LOC108783645	rs1800730	GSA
NM_000410.3(HFE):c.845G>A (p.Cys282Tyr)	HFE	rs1800562	GSA
NM_000031.5(ALAD):c.823G>A (p.Val275Met)	ALAD	rs121912981	GSA
NM_000031.5(ALAD):c.718C>T (p.Arg240Trp)	ALAD	rs121912982	GSA
NM_000031.5(ALAD):c.397G>A (p.Gly133Arg)	ALAD	rs121912980	GSA
NM_000031.5(ALAD):c.36C>G (p.Phe12Leu)	ALAD	rs121912984	GSA
NM_000375.2(UROS):c.683C>T (p.Thr228Met)	UROS	rs121908014	GSA
NM_000375.2(UROS):c.673G>A (p.Gly225Ser)	UROS	rs121908020	GSA
NM_000375.2(UROS):c.244G>T (p.Val82Phe)	UROS	rs121908016	GSA
NM_000375.2(UROS):c.217T>C (p.Cys73Arg)	UROS	rs121908012	GSA
NM_000375.2(UROS):c.184A>G (p.Thr62Ala)	UROS	rs28941775	GSA
NM_000375.2(UROS):c.10C>T (p.Leu4Phe)	UROS	rs121908015	GSA
NM_000190.4(HMBS):c.445C>T (p.Arg149Ter)	HMBS	rs118204120	GSA
NM_000190.4(HMBS):c.499C>T (p.Arg167Trp)	HMBS	rs118204101	GSA
NM_000190.4(HMBS):c.500G>T (p.Arg167Leu)	HMBS	rs118204095	GSA
NM_000190.4(HMBS):c.500G>A (p.Arg167Gln)	HMBS	rs118204095	GSA
NM_000190.4(HMBS):c.601C>T (p.Arg201Trp)	HMBS	rs118204109	GSA
NM_000190.4(HMBS):c.606G>T (p.Val202=)	DPAGT1|HMBS	rs1131488	GSA
NM_000190.4(HMBS):c.1075G>A (p.Asp359Asn)	HMBS	rs144949995	GSA
NM_001382.3(DPAGT1):c.1177A>G (p.Ile393Val)	DPAGT1|HMBS	rs643788	GSA
NM_001382.3(DPAGT1):c.994T>G (p.Phe332Val)	DPAGT1|HMBS	rs138544311	GSA
NM_000374.4(UROD):c.603A>G (p.Pro201=)	UROD	rs2228084	OmniExpress
NM_000309.4(PPOX):c.-186C>A	PPOX	rs2301286	OmniExpress
NM_000410.3(HFE):c.187C>G (p.His63Asp)	HFE|LOC108783645	rs1799945	OmniExpress
NM_000190.3(HMBS):c.-65C>T	HMBS	rs589925	OmniExpress
NM_000190.4(HMBS):c.88-14G>A	HMBS	rs17075	OmniExpress
NM_000190.4(HMBS):c.613-19C>A	HMBS	rs1784304	OmniExpress
NM_001382.3(DPAGT1):c.*427T>G	DPAGT1|HMBS	rs28990975	OmniExpress
NM_001382.3(DPAGT1):c.*417T>C	DPAGT1|HMBS	rs7759	OmniExpress
NM_001382.3(DPAGT1):c.*265A>G	DPAGT1|HMBS	rs28990974	OmniExpress
NM_001382.3(DPAGT1):c.1177A>G (p.Ile393Val)	DPAGT1|HMBS	rs643788	OmniExpress

When people with porphyria access UPWARD, they are met with a survey built to collect consent, contact information and disease-associated information, such as clinical symptoms, genetic and environmental data, including targeted questions concerning environmental factors suspected to trigger acute porphyria attacks. Participants are given the option to share this survey with family members and friends, both those with and without porphyria symptoms. Although family members and friends without symptoms at the time of the survey will likely never develop symptoms (due to the low penetrance of porphyria-associated mutations), we seek to identify modifying genes and environmental factors that contribute to the phenotype through comparing genotypes of these individuals with those of people reporting latent and active porphyria ^64^. We plan to explore the possibility of recruiting participants by adding UPWARD links to the SNPedia research database, as well as through collaborating with porphyria advocacy and patient-education groups, and clinical partners.

### Project 2: Variants Discovery and Rapid Clinical Diagnosis

**Figure 2.**
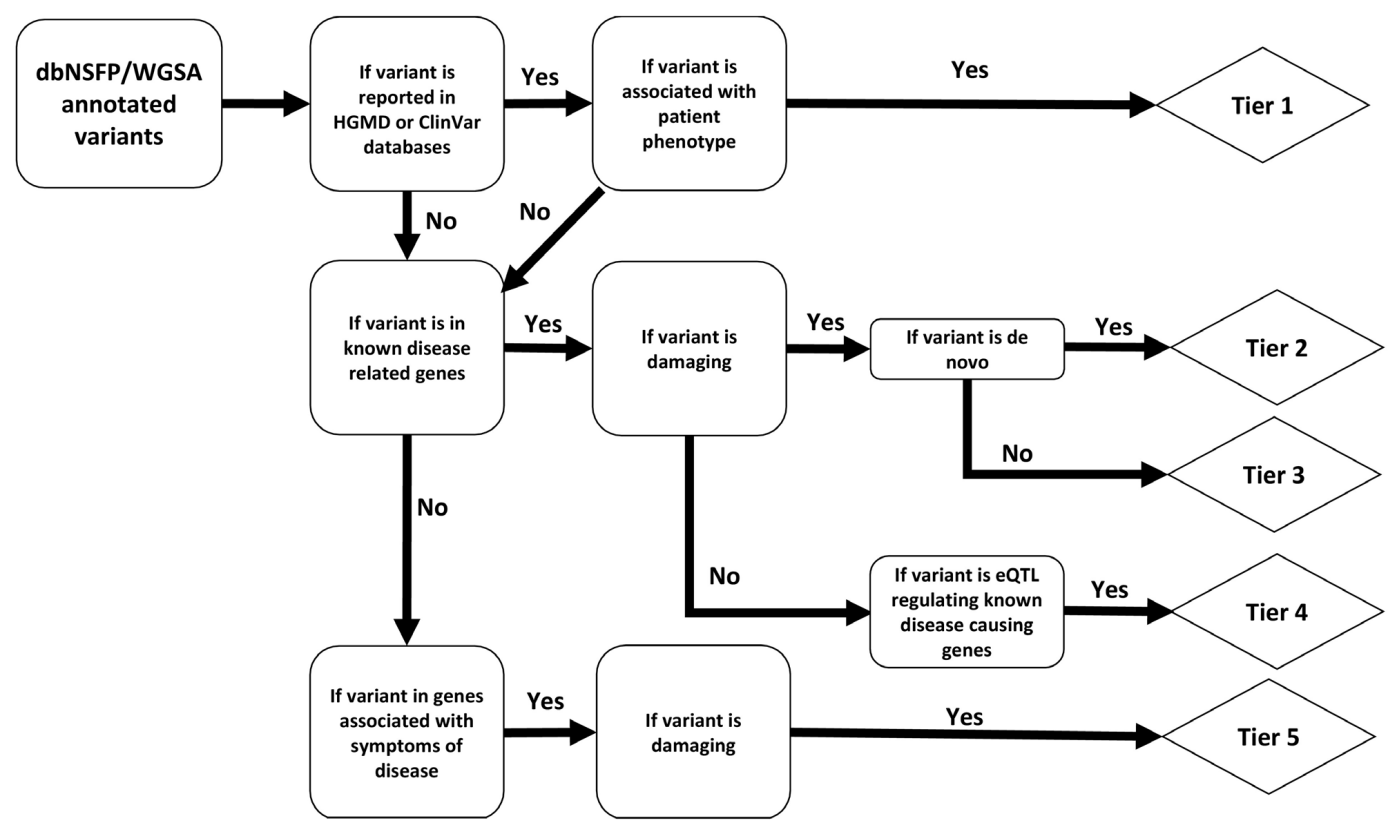
Overview of the Variants Discovery pipeline to report possible pathogenic variants associated with Mendelian diseases. Abbreviations: dbNSFP, database for nonsynonymous SNPs’ functional predictions; WGSA, whole genome sequencing annotator; HGMD, Human gene mutation database; eQTL, expression quantitative trait loci.

Many different mutations can contribute to the onset and progression of porphyria ^65^. We designed a method to search for underlying genetic variants associated with symptoms of congenital erythropoietic porphyria (CEP). The first diagnostic steps to confirm CEP often happen after referral to a genetic counsellor, who recommends targeted screening for a panel of known-pathogenic porphyria-associated SNPs. In cases where no known-pathogenic variants are found, whole-exome sequencing may be recommended of both the patient and their parents to catalog variation in the symptomatic person versus their asymptomatic parents. These variants filtered from the parent-child “trio” data can then be annotated with available disease-associated information, if any, using existing tools (such as dbNSFP and WGSA) ^66, 67^. With our Variants Discovery tool, we aimed to generate a workflow which operates on trio-data to identify, categorize and then rigorously assess candidate disease-causing mutations in cases where the underlying mutation is unknown, modeled after existing workflows for whole-exome sequence analysis ( Figure 2) ^68^.

Tier 1 variants are known disease-causing mutations in known disease-causing genes. Tier 2 variants are uncharacterized *de novo* mutations predicted to be damaging (see Methods) in known disease-causing genes. Tier 3 variants are uncharacterized, damaging, inherited mutations in known disease-causing genes (parents are not affected). Tier 4 variants are functional mutations with unknown significance in known disease-causing genes. Tier 5 variants are damaging mutations in the extended gene list (e.g. those genes associated with symptoms of disease). Candidate disease-causing variants are categorized into five evidence-based tiers, where Tier 1 variants are known-pathogenic and have the highest support. We intend to expand this workflow so that it might be used to assist in the diagnosis of patients with other difficult-to-identify conditions.

### Project 3: MassiveSeq: Automated meta-analysis of RNA-Seq Data from GEO data

The fields of biology and medicine have undergone swift changes to the manner in which ribonucleic acid (RNA) can be studied using deep-sequencing techniques to investigate expression-differences in possible RNA species that may be associated with deleterious disease outcomes ^69^. RNA-Seq technology has revolutionized detection and analysis of aberrant RNA transcripts associated with disease ^69^.

In rare disease research in particular, obtaining sample-sizes enabling confident identification of disease-associated transcripts is a considerable challenge. The amount of RNA-seq data contributed to NCBI’s Gene Expression Omnibus (GEO), a public repository for functional genomics data, is increasing at a rapid pace. A simple query for “Expression profiling by high throughput sequencing” yielded 14,200 unique datasets as of March 6, /2019. The availability of these massive quantities of data creates an open opportunity in many research areas for meta-analyses using these published datasets to strengthen analytical power. Our massive parallel-sequencing analysis tool, MassiveSeq, provides an opportunity for researchers and bioinformaticians to easily extract and process meaningful information (such as quantitative gene expression, novel transcripts and their isoforms, alternative splice-site variants, SNPs or copy-number variation) from these large datasets to evaluate the associations between biological processes, gene expression and disease outcomes. MassiveSeq automates downloading and processing of large-scale RNA-seq datasets with the aim of easing computational time and complexity ^70– 73^.

**Figure 3.**
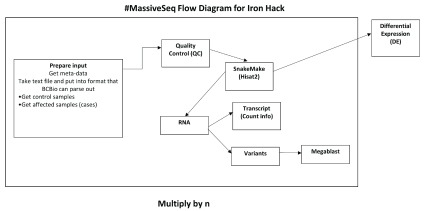
Flowchart for Massiveseq Methodology. The pipeline takes metadata from the Sequence Read Archive (SRA) and parses it for quality control (QC). The primary work takes place in a custom snakemake script that aligns sequences with Hisat2 and then quantifies transcripts with Stringtie in a parallelized fashion across available machines and cores.

MassiveSeq differs from conventional, comprehensive RNA-seq pipelines in that it combines multiple RNA-seq datasets to increase analytical power ( Figure 3) ^74– 76^. The search is confined to samples meeting the criteria, e.g., disease, library source (genomic, transcriptomic or metagenomic), platform (Illumina, PacBio), or instrument (Genome Analyzer, Hiseq, Nextseq). MassiveSeq additionally allows exploration of novel clustering methods to enable meta-analysis of differential gene expression. Initial steps in processing raw sequencing reads on even a single, traditional dataset are often computationally intensive, and obtaining additional publicly-available RNAseq datasets at a massive scale for such processing is resource-consuming as well. MassiveSeq takes raw-sequencing data (fastq format) automatically streamed from NCBI’s Short-Read Archive (SRA) as input, using a GEO query specifying parameters such as disease and experimental type (e.g., high-throughput RNAseq). Datasets can be further filtered as needed. The MassiveSeq pipeline next utilizes dockerized HISAT2 (version 2.1.0) and StringTie (v1.3.5) to enable automated, parallel processing of each experiment ^77, 78^. Reads are automatically streamed directly from SRA, mapped to a reference genome, assembled into transcripts--including novel splice-variants--and annotated in parallel within each dataset. The MassiveSeq pipeline allows uniform processing of multiple, independent RNAseq datasets, enabling powerful identification of differentially expressed genes and transcripts associated with diseases of interest. We applied MassiveSeq to 99 Friedreich’s Ataxia SRA datasets to identify disease-associated transcripts for Iron Hack ^70– 73^.

### Project 4: Phenogeno Viz: Rapid aberrantly-expressed gene identification from RNA-Seq

**Figure 4.**
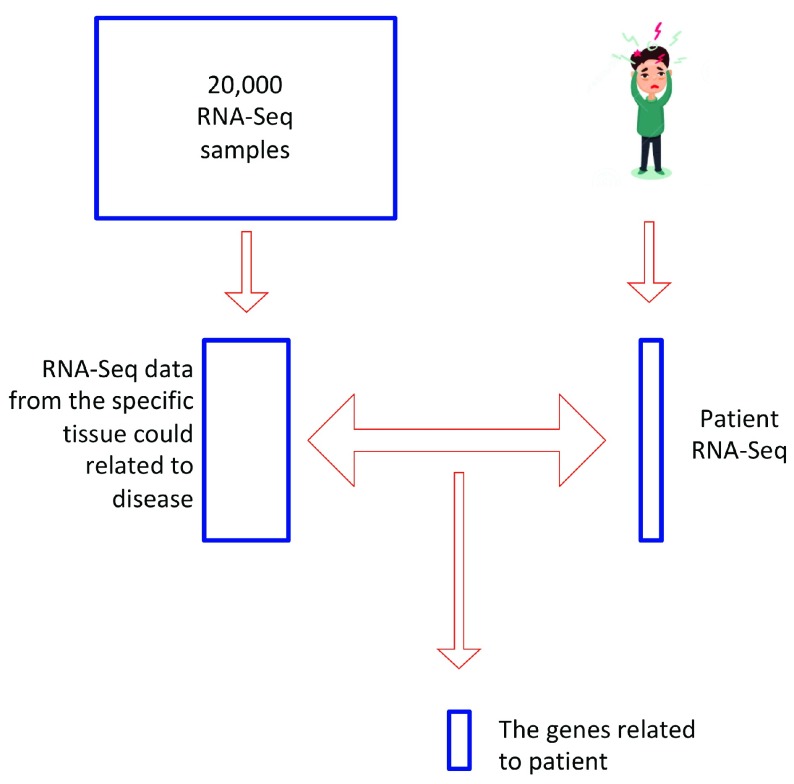
The work flow chart for identifying abnormal genes based on RNA-Seq. After RNA-Seq is performed on a patient sample, the program searches the Genotype-Tissue Expression Project (GTEx) database for RNA-Seq data from the specific tissue potentially associated with the disease. Three methods are used for RNA-Seq normalization (Fragments per kilobase of transcript per million mapped reads (FPKM), transcripts per million mapped reads (TPM) and Differential gene expression analysis based on the negative binomial distribution (as implemented in DESeq)), and the data were fit to a Gaussian mixture model to remove noise within samples. The differentially expressed genes in the patient sample are finally captured by using the R program DESeq.

Abnormal gene-expression patterns can cause a broad range of diseases. However identifying abnormally-expressed genes and correctly interpreting expression data across experiments can be complicated by inconsistencies in gene-expression normalization strategies, as well as inadequate filtering of noisy data. Here, we developed an algorithm to rapidly identify genes with abnormal gene expression patterns in samples of interest (e.g., disease-presenting patient) as compared to controls ( Figure 4). This method was built utilizing ~2000 RNA-Seq datasets publicly available on GTExPortal ^79^. The package implements three commonly used RNA-Seq normalization methods: Fragments per kilobase of transcript per million mapped reads (FPKM), transcripts per million mapped reads (TPM) and differential gene expression analysis based on the negative binomial distribution (DEseq). A Gaussian-mixture model is utilized here to remove RNA-Seq noise and the DE-Seq method is finally implemented to capture abnormally expressed genes corresponding to query tissue. Simulation data were generated to test algorithm performance, and we intend to expand this system so that it might be used to assist in the diagnosis of patients with difficult to identify conditions ^80^.

### Project 5: Phenotype-to-Genotype Mapping: Assessing combinatorial variant-contribution to disease phenotypes

Disease-phenotypes are unlikely to be entirely explained by the presence of single pathogenic variants. Pleiotropy, modulating genes and combinatorial effects are the rule, rather than the exception; however assessing combinatorial effects underlying disease quickly becomes computationally expensive, with a practically-infinite number of variant-combinations that could be assessed. We developed a tool-set to enable thoughtful reduction of variants to feasibly assess the role of modifying genes in rare diseases such as Friedreich’s ataxia.

Most of the alleles a person inherits are unlikely to be involved in modulating the disease phenotype, and models incorporating many extraneous variables are unnecessarily cumbersome and perform more poorly than models incorporating domain-specific feature-selection. Therefore the first step of our pipeline was to reduce the disease-associated variant search-space to genes fitting a profile of interest.

We focused on broadly-applicable features of likely disease-causing variants (as opposed to disease-specific features) for our first layer of feature-selection in this first iteration of our pipeline. Input variant-call data are filtered based on the likelihood that any particular variant is deleterious (as predicted by Polyphen-2 scores) and by residual variation inheritance scores ^81, 82^. As features-of-interest for disease-associated variants should change depending upon the particular disease, phenotype, and available domain-specific knowledge, the feature-selection component of our tool is intended to be easily extendable for investigating the combinatorial contributions of multiple variants to disease phenotypes by any number of characteristics. We incorporated two existing annotation packages (Open- CRAVAT (version 1.4.0) and ANNOVAR version (On 2018Apr16)) to thoroughly annotate available information for each variant, any of which can be filtered on in the feature-selection module ^83^. Highly ranked variants are then assessed for their contribution to disease-phenotype via the equally modular “analysis” part of our pipeline. Our current analysis module utilizes the APRIORI algorithm to detect variant co-occurrence relationships with disease, though the output from the feature-selection module is in a common format to facilitate application of other machine-learning approaches to identifying combinatorial interactions, all implemented via a simple web-user interface ^84^.

We developed this pipeline with modularity being a primary goal. The APRIORI algorithm is currently implemented to identify genes that frequently co-occur in the feature-selected set of genes. Future work will implement tools that check for over-representation of gene ontology terms among the genes determined to have deleterious alleles.

## Methods and implementation

Key concepts informing methods and implementations of each project are described below.

### UPWARD

To build a database of pathogenic or likely pathogenic SNPs, we sourced Rapid Stain Identification Series (RSID) information from the Illumina OmniExpress & Illumina Global Screening Array (GSA) microarray chips (used by Ancestry and 23andMe respectively), then filtered out non disease-associated genes using the NCBI ClinVar database. For specific application to porphyrias, we selected all genetic polymorphisms annotated to be associated with any of the porphyrias, as well as their associated RSID, SNP location in the genome, and degree of pathogenicity ^85, 86^. Participants’ raw genomics data and environmental data are stored in a non-relational database, which has been proven to be more efficient than relational databases for storing and accessing genomic data ^87, 88^. A secure, HIPAA-compliant human subject meta-information database will be built as part of the next iteration of development ^89, 90^. A secure, HIPAA-compliant human subject meta-information database will be built as part of the next iteration of development ^89, 90^. At that time, the database will be expanded to capture the following information: 1) patient-reported phenotype and symptom information of people identified as potentially carrying a pathogenic or likely pathogenic variant in a porphyria gene and 2) people with a clinical diagnosis of porphyria, as well as de-identified information on their family members to try to capture data on asymptomatic people.

Our system currently consists of a cloud-database built on MongoDB Community Edition, and a web server run through NGINX to accept input data from participants. The entire system is containerized and orchestrated by Docker Compose for ease of replication and to enable application to other diseases.

### Variants Discovery and Rapid Clinical Diagnosis

Our pipeline categorizes patient variant-data into five tiers of pathogenic certainty based on quality of evidence, the logic of which is broadly outlined in Figure 2. The pipeline accepts dbNSFP or WGSA-annotated patient variant-files (in tab-delimited format, one variant per line). Annotated variants are first checked against known disease-associated variant databases, namely HGMD and ClinVar, to identify any previously reported pathogenic mutations matching the patient phenotype; these known, disease-causing variants in known disease-causing genes are categorized into the most confident classification, Tier 1. All variants not represented in the HGMD and ClinVar databases are next checked to see if they are located in genes that are involved in known disease-associated pathways. Variants in disease-associated pathways are then evaluated for probability of being deleterious (with start-loss, stop-gain, essential splicing variant, frameshift, indel or missense-mutations being highly likely to be deleterious). Damaging variants in known disease-causing genes are then contrasted against variant files from the non-affected parents to distinguish *de novo* (Tier 2, uncharacterized, damaging *de novo* mutation in known disease-causing gene) from inherited (Tier 3, uncharacterized, damaging inherited mutation in known disease-causing gene) variants. Non-deleterious variants are not considered further.

Damaging variants not occurring in known disease-causing genes themselves, but mapping to known expression-Quantitative Trait Loci (eQTLs, loci associated with expression-changes in transcripts from known disease-causing genes), are stratified into Tier 4. We report all other damaging variants in or associated with genes that are related to symptoms of the disease as Tier 5 (damaging mutation in the extended gene list). Any other known disease-causing mutations associated with unrelated diseases are additionally reported in an extended report to allow for possible incidental or secondary findings.

### MassiveSeq

We planned the main snakemake (version 5.4.2) to automate dispatching of jobs depending on the available cores and memory of a machine ^91^. Here, the core steps involved Hisat2 for alignment, followed by Stringtie (v1.3.5) for transcript annotation and *de novo* annotation. Finally, reads were quantified by using featureCounts to measure at the exon level from the subRead (version 1.6.3) package ^92^. This quantification pipeline follows a common, recently published protocol on Stringtie and Hisat2 ^93^. It allows for both known as well as novel isoform transcripts to be identified and measured.

Once the gene counts were fully quantified for each sample, we analyzed the overall dataset comprised of all 4 studies using the R package metaSeq (version 1.22.1) ^94^. This package adapts the non-parametric NOISeq method for differential RNA-seq analysis to allow for multiple studies in a meta-analysis framework ^95^.

We used the gene counts with GSVA (Gene Set Variation Analysis, version 1.30) to estimate per-same GSEA (Gene Set Enrichment Analysis) pathway enrichments for the 50 hallmark datasets from MSigDB ^96, 97^. We used these pathway enrichments as features (a binary up- or down-regulated pathway) for a deep learning model, along with the remaining gene estimates. We used the fast.ai library to construct a Convolutional Neural Network (CNN) ^98^. One of the novel features of fast.ai, especially for our data, is that it facilitates rapid construction of neural networks with tabular data via embeddings similar to Word2Vec ^99^. The training split was 70/30; afterwards the CNN was trained for 5 epochs (cycles of the data), with a learning rate of 0.1.

We are also in the early stages of adapting the GATK RNA-seq best practices to this pipeline so that we can rapidly call variants on these samples ^100^. Our workflow for the procedures, and methods used can be found in Figure 3.

### Phenogeno Viz

This package is designed to detect abnormal genes exhibited differential expression compared with normal tissue cell. For each patient RNA-Seq result, we first download the gene expression level from normal tissue same as patient tissue. Then for multiple gene expression RNA-Seq samples available in website GTExportal ( https://gtexportal.org/home/), three normalized methods including TPM, FPKM and DEseq are available. The Gaussian-mixture model is utilized to remove RNA-Seq noise. The basic idea is to use the EM algorithm to find two best fitted Gaussian distribution and only maintain the distribution with relative higher mean value as a true signal. After noise reduction, DEseq algorithm is used to identify significant up or down regulated genes in patients.

### Phenotype to Genotype-Mapping

**Figure 5.**
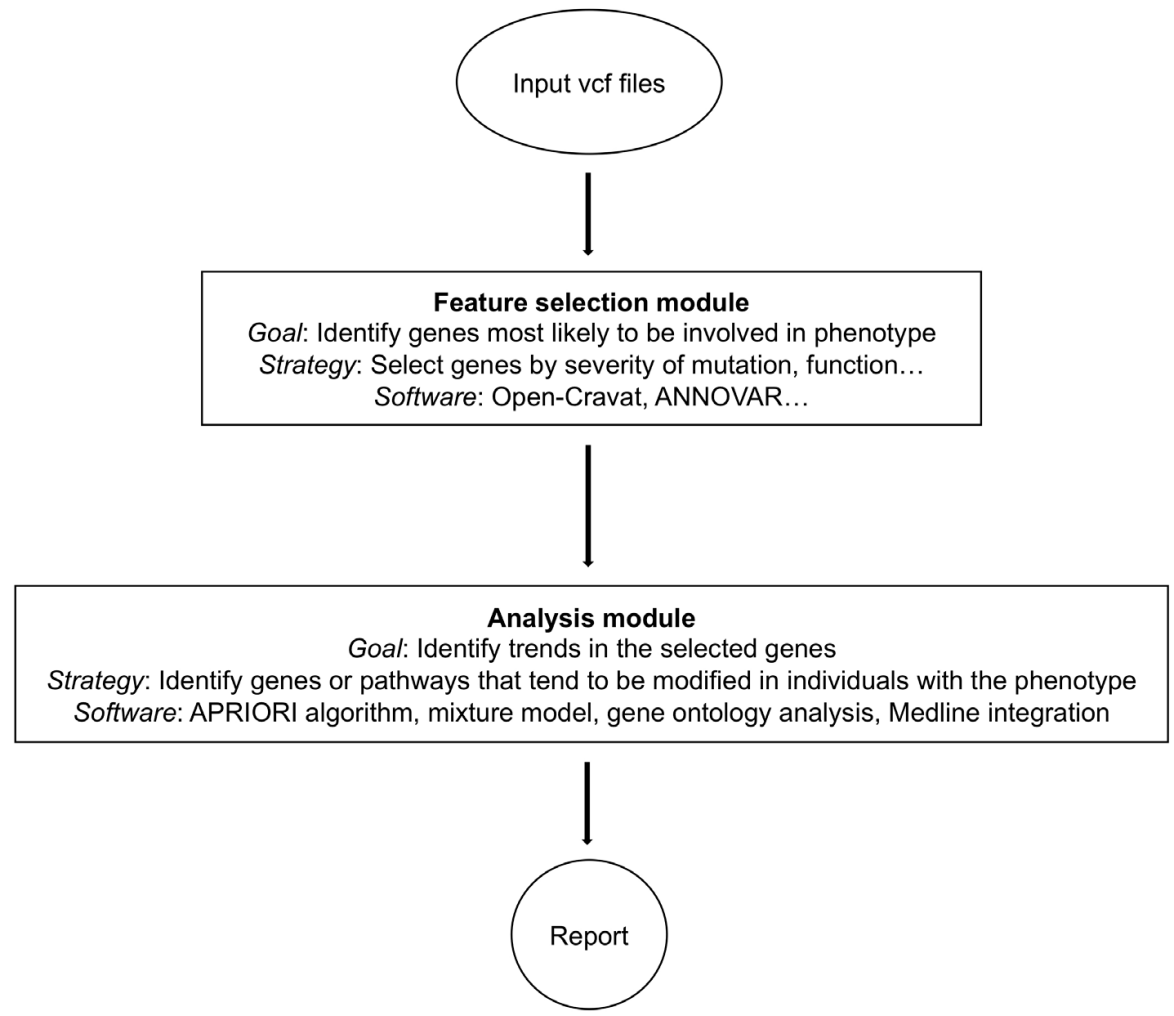
Phenotype-to-Genotype Mapping: Assessing combinatorial variant-contribution to disease phenotypes general workflow. Input data are variant-call files in .vcf format collected from patient samples. The feature-selection module collects all available annotation information for each identified variant, then narrows down to variants most likely to be associated with the phenotype based on user-specified parameters. These feature-selected variants are then analyzed for combinatorial contribution to the disease using the tools in the analysis module. The output of the analysis modules are tables and graphs that summarize the results.

This tool is envisioned to be most useful for analyzing variant-call data from networks of families affected by rare disease ( Figure 5). Currently the input data are variant-call files (.vcf) obtained from patients with a rare disease; future versions will incorporate additional genomic information from family members. The user uploads patient .vcf files through a web-app interface, then selects features on which to filter (currently a maximum and minimum score for residual variance-intolerance, a measure of gene-tolerance to variation based on population allele-frequencies). The web interface can be run locally to keep patient data secure. The back-end next runs the files through CRAVAT and ANNOVAR to assemble annotation information on all variants compiled from multiple databases that the user selects upon install (including ClinVar, Pubmed, etc). These annotation data are then filtered according to user-specification using the feature selection module.

## Operation

Operation can be performed on a computational cluster with multiple cores. The system can use a Lustre parallel file system for fast Input and Output. Remote mounting onto the cluster should be available for flexible data access and movement.

### UPWARD

The only requirement to build this system is having Docker and Docker Compose installed on your machine. For instructions on running the system refer to the associated GitHub readme at bit.ly/UPWARD19 ^101, 102^.

### Variants Discovery and Rapid Clinical Diagnosis

GitHub readme and description available at https://bit.ly/2FGqkv7 ^103, 104^.

### “Massive” RNA-seq Combined Analysis of Multiple Datasets

For full instructions on how to clone and implement the code, please refer to:

The MassiveSeq github repository: https://bit.ly/2HKA61y ^105, 106^.

### PhenogenoViz: Rapid abnormal gene identification based on RNA-Seq

Running the web app requires the installation of Ruby, R, and Python on the server. The instructions for installing Ruby on Rails on Windows 10, Ubuntu, and OS X can be seen here, and should be similar for different OS versions ^107^. Install Python 3 from here and install R from here ^108, 109^. The web application is available on GitHub at https://bit.ly/2V3Hpo2 and instructions for installation are detailed on the ReadMe ^110, 111^.

### Phenotype to Genotype-Mapping

GitHub readme and description available at https://github.com/NCBI-Hackathons/pheno_geno_ataxia ^112, 113^.

## Lessons learned

Throughout this process we identified several areas where improvements could be made for future disease-focused hackathons. A few of these are described below.

1) We were successful in prototyping for a specific disease.
2) It was helpful to learn more about the diseases and current problems that need to be solved before starting the projects.
3) If trying to solve a clinical problem, such as how to improve and speed up the rate at which patients receive a diagnosis for rare diseases, include clinicians as part of the group.
4) It may also be advantageous to have the didactic presentations about the diseases in advance of the hackathon such that everyone has a basic understanding of the issues and disease symptoms and time for brainstorming.
5) Having a team meeting prior to the hackathon to assign roles and discuss overall flow for each day was helpful.
6) Providing literature to read about the disease/genetic condition was also useful background for preparing for the hackathon.
7) Having two leads on each team increased efficiency, as each could take turns fielding questions from less-experienced team-members while the other could keep the hacking on-task for the day.
8) A few things some specific teams learned
  a) UPWARD: Forming a team composed of people with a variety of training backgrounds (e.g. clinicians, researchers, organizers, computer scientists, biologists, geneticists, etc.) brings strength and utility to team ideas and project results. Additionally devoting a portion of the first day or meeting period to brainstorming, idea proposal, and arguments facilitates the formation of a plan which team members are able to agree upon and work towards while reducing the chance of a schism further down the road.
  b) MassiveSeq: We initially overscoped/planned on a completely different toolset. Scaling back to a core set of tools that we were comfortable with made completing the project feasible. People were coming in from really different backgrounds (choice of programming language, familiarity with genomics data formats, etc.) and in retrospect we would have liked to have planned a bit more for some specific tasks.

## Results

### UPWARD

After identifying porphyria-related pathogenic SNPs currently included on Illumina OmniExpress and Illumina GSA microarray chips, 43 porphyria-related pathogenic SNPs were found. This list (presented in Table 1) will be maintained and updated at the UPWARD GitHub repository (labeled as pathogenic SNPs.csv) ^101^. This list will be compared against participant-submitted consumer genomics test results within UPWARD once the project is reviewed and approved by the Institutional Review Board (IRB).

### Variants discovery and rapid diagnosis

**Figure 6.**
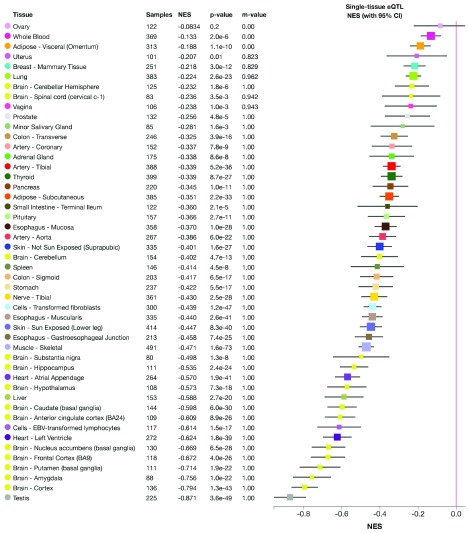
Expression change of the UROS gene caused by eQTL SNP No. 1 across all tissue types in the Genotype-Tissue Expression Project (GTEx). There is significant down-regulation of UROS gene associated with this variant in all tissues (except ovary). NES: normalized effect size.

**Figure 7.**
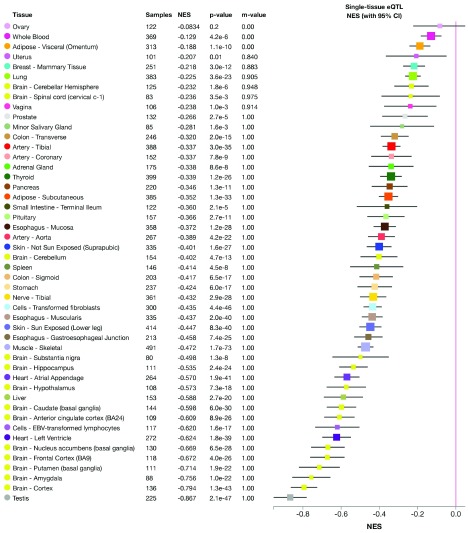
Expression change of the UROS gene caused by eQTL SNP No. 2 across all tissue types in the Genotype-Tissue Expression Project (GTEx). There is significant down-regulation of UROS gene associated with this variant in all tissues (except ovary). NES: normalized effect size.

We examined the genetic basis with the following examples of porphyria genetics. Using our developed pipeline, we successfully identified several candidate SNPs (in Tier 1, Tier 2 and Tier 4) that were previously unnoticed in a porphyria patient in a clinical setting. These SNPs are located in genes known to cause different kinds of porphyria, e.g. UROS and CPOX genes. The discovered SNPs can, based on the prediction using our pipeline, affect the transcription of the candidate genes, their translation or both. All of these possibilities would result in functional abnormalities of the final gene product. For example, two of the identified SNPs were eQTL (expression quantitative trait loci), which led to significantly decreased expression level of the UROS gene ( Figure 6 and Figure 7).

These variants examples demonstrated that our pipeline can help physicians and/or clinical geneticists quickly filter out the vast majority of neutral variants and report the remaining variants in clinically meaningful tiers to facilitate further experimental validation and explanation.

### MassiveSeq

**Figure 8.**
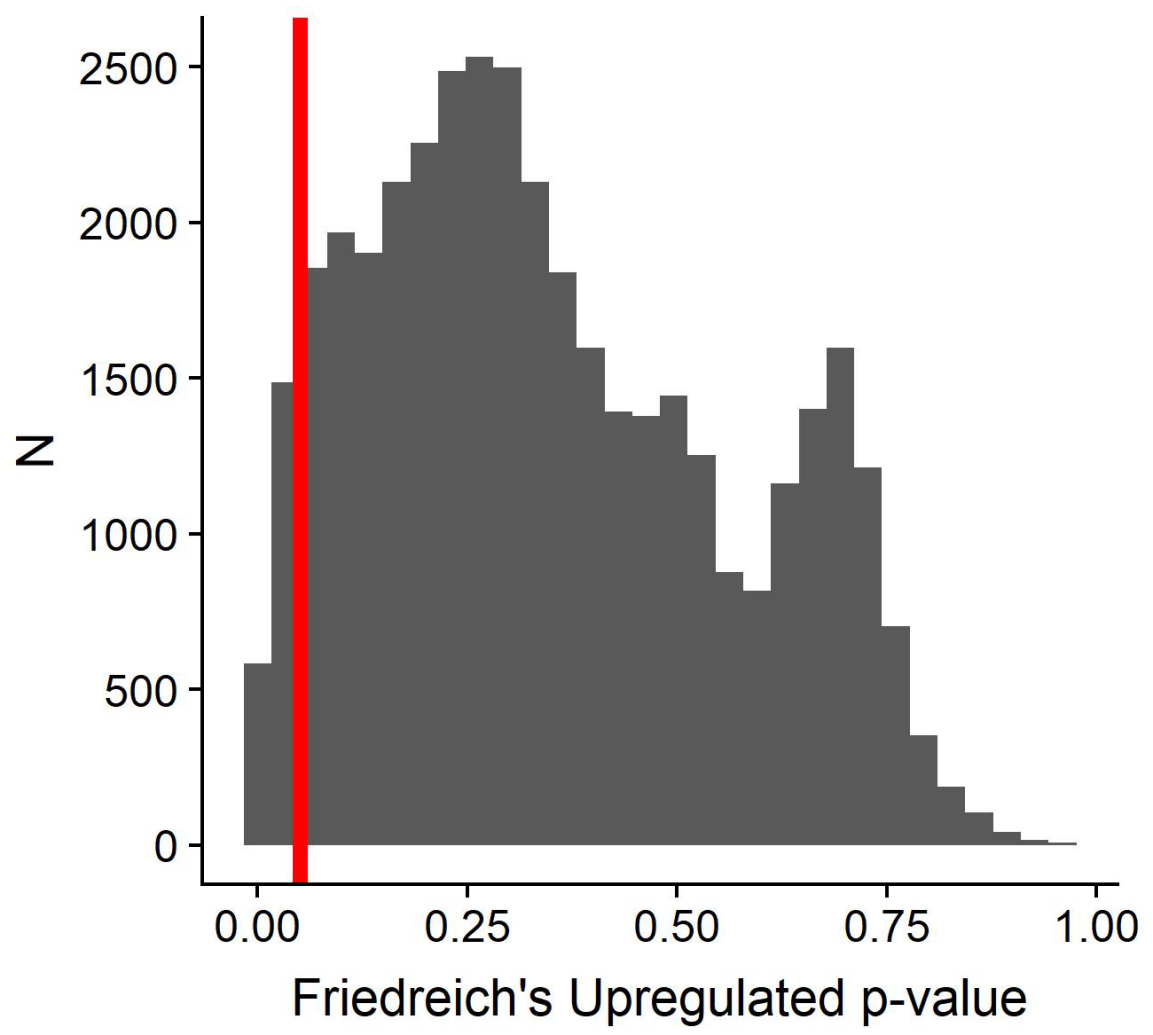
Significance of up-regulated genes from metaseq analysis; red bar denotes .05 significance cutoff. Distribution of significance in downregulated genes from metaseq analysis; no genes were significant at 0.05 threshold.

Using Metaseq we identified over 2000 genes upregulated in Friedreich’s Ataxia patients compared to controls ( Figure 8). However, we emphasize that this analysis was merely a proof of concept, and further work needs to be done to explore methods and techniques for standardizing phenotypes (data harmonization) alongside the meta-analysis itself in Friedreich’s Ataxia. ( Figure 8).

We used fast.ai to train a CNN on an embedded feature-space of these counts as well as 50 gene-set enrichment features from Msigdb (see Methods). The trained model had an overall accuracy of 0.75, which seems promising given the number of features and low number of samples for training.

MassiveSeq’s implementation of HISAT2 and StringTie identified novel-isoform transcripts in various samples. We focused our analyses on the FXN gene, as trinucleotide GAA-repeats at this locus are causative of FRDA. We identified multiple novel-isoform transcripts within 1kb up and downstream of FXN in affected, unaffected and carrier-patients ( Table 2). We were able to visualize the truncation of the FXN transcripts from the above samples using IGV. Shallow read-coverage of the whole transcriptome from this particular study made it difficult to confirm the reliability of the identified transcript truncation.

**Table 2.** List of novel-isoform transcripts within 1kb of the FXN gene.

Novel Transcript	Chr.	Strand	Start	End	FPKM	TPM	Disease
SRR8038380_chr.30572	9	+	69035259	69100178	1.787076	3.825795	Friedrich Ataxia
SRR8038380_chr.30573	9	-	69107926	69108217	0.136139	0.291447	Friedrich Ataxia
SRR8038387_chr.17699	9	+	69035259	69079076	0.274068	0.490055	Carrier
SRR8038389_chr.19844	9	+	69035751	69074850	1.070571	1.139182	Unaffected
SRR8038390_chr.21253	9	+	69035259	69100178	1.033484	1.298192	Unaffected
SRR8038399_chr.26427	9	+	69035259	69079076	3.126959	7.802162	Unaffected

### Phenogeno Viz: Rapid abnormal gene identification based on RNA-Seq

For each input patient RNA-Seq data, the RNA-Seq data related to query tissue are extracted from the database. The available tissues and number of RNA-seq data are listed in Table 3.

**Table 3.** Available RNA-Seq data samples in Genotype-Tissue Expression Project (GTEx) for different tissues.

Tissues	Number of RNA-Seq data
Adipose	797
Adrenal	190
Bladder	11
Blood	536
Blood	913
Brain	1671
Breast	290
Cervix	11
Colon	507
Esophagus	1021
Fallopian	7
Heart	600
Kidney	45
Liver	175
Lung	427
Muscle	564
Nerve	414
Ovary	133
Pancreas	248
Pituitary	183
Prostate	152
Salivary	97
Skin	1203
Small	137
Spleen	162
Stomach	262
Testis	259
Thyroid	446
Uterus	111
Vagina	115

**Figure 9.**
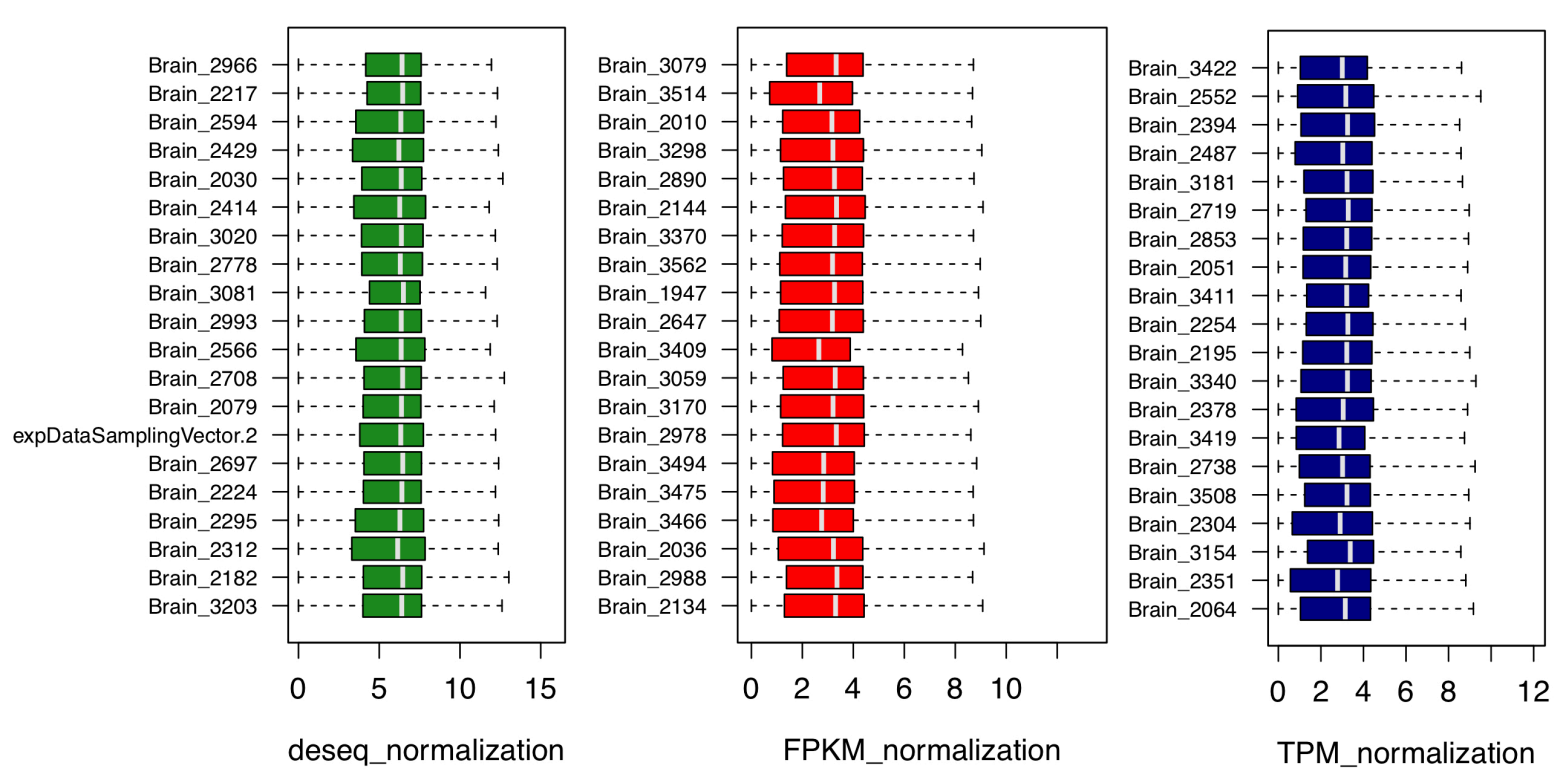
n = 20 genes are sampled here to compare different normalization method: Fragments per kilobase of transcript per million mapped reads (FPKM), transcripts per million mapped reads (TPM) and Differential gene expression analysis based on the negative binomial distribution (DESeq).

**Figure 10.**
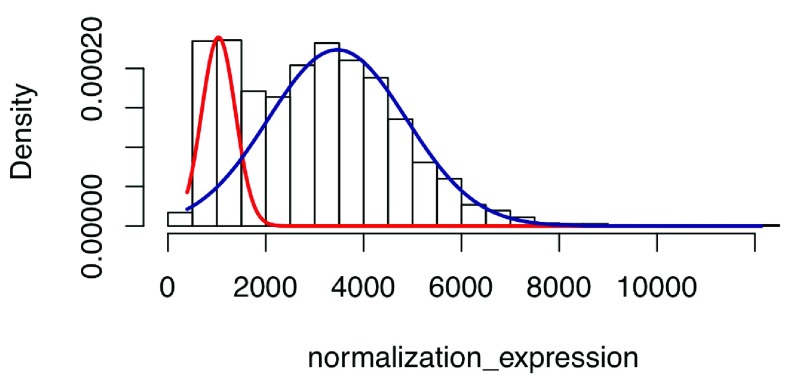
The Gaussian mixture model is implemented here to filter out noise. Hist plot shows the distribution of gene expression level for gene ‘CELSR2’ in 1671 different brain RNA-Seq samples. The Gaussian mixture model is fitted by the EM algorithm and the noise is filtered out by posterior probability bigger than 0.5.

**Figure 11.**
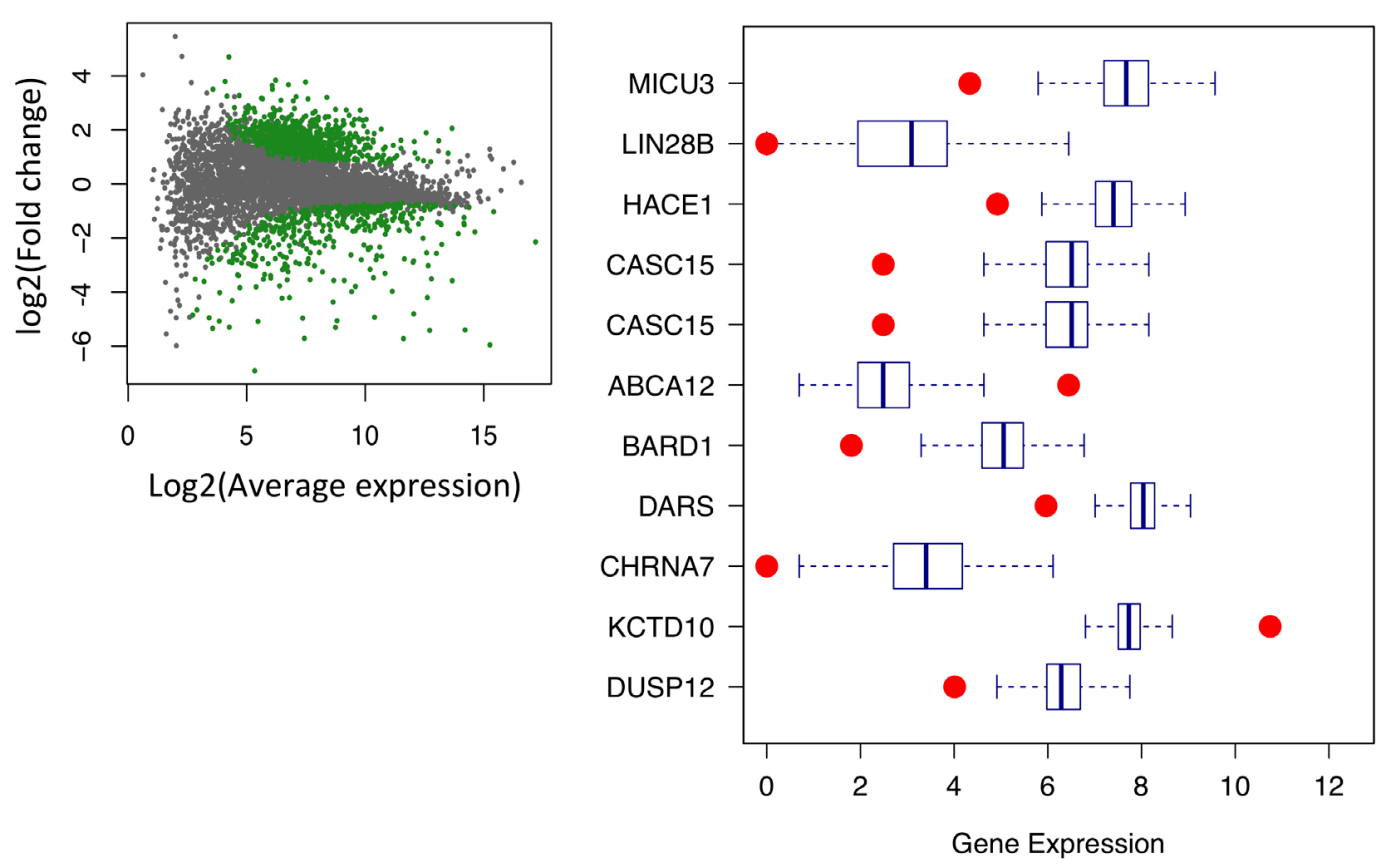
Differential gene expression analysis based on the negative binomial distribution (DESeq) is used here to find differential expression genes between patient and database. **A**) Scatter plot shows significant differential genes (green dot, p-adj < 0.01). **B**) Boxplot shows top 10 abnormal genes in simulation compared with data from database.

For convenience, brain tissue is selected here for the following discussion. Considering different sequencing depth for each sample, we provide three methods for data normalization: DEseq, FPKM, and TPM. As there are a large number of samples, we used uniform sampling to select n genes for visualization. As shown in Figure 9, n = 20 genes are shown here to compare different normalization methods. DEseq normalization results show relative lower fluctuation compared with the other two methods (F-test p < 2.2e16), indicating better performance of DEseq. Except for Bladder, Cervix, and Fallopian, most tissues in our database exhibit large RNA-Seq sample number. Therefore, a method is required to select the data with relatively high signal/noise ratio. A Gaussian-mixture model is fit for each gene and returns the posterior probability to be ‘true’ signal for each RNA-Seq sample ( Figure 10). The top ten samples with the highest average posterior probability are picked as background and compared with patient samples. Then, DEseq is used for differential expression gene identification. As shown in Figure 11A, the green dots represent significant differential expression gene (p-adj < 0.01) between patient and samples from database. Figure 11B shows top 10 abnormal genes and their geneID in patients.

(‘Neuroblastoma’ related genes is used here).

### Phenotype-to-Genotype Mapping

The code was tested using the related individuals from the 1000 genomes project. Flagging the genes most likely to have deleterious alleles decreased the search space enough to allow the APRIORI algorithm to run on the dataset.

## Conclusion and next steps

Common questions in the community about hackathons include whether they can focus on specific diseases and how clinical personnel can interact more effectively with data scientists. We found that it was indeed possible to focus on a given disease while developing generalized tools in a hackathon. In fact, we found it helpful to have cases to use in our analyses from a specific disease. Finally, we found it was to the benefit of everyone to have clinical personnel involved, especially in the later stages of the event.

## Software availability

### UPWARD

Source code: https://github.com/NCBI-Hackathons/UPWARD

Archived source code: http://doi.org/10.5281/zenodo.3236567 ^102^

License: MIT

### Rapid Clinical Diagnostics

Source code: https://github.com/NCBI-Hackathons/Rapid_Clinical_Diagnostics

Archived source code: http://doi.org/10.5281/zenodo.3236563 ^104^

License: MIT

### MassiveSeq

Source code: https://github.com/NCBI-Hackathons/MassiveSeq/

Archived source code: http://doi.org/10.5281/zenodo.3236565 ^106^

License: MIT

### PhenoGeno Viz

Source code: https://github.com/NCBI-Hackathons/Phenogeno_Viz

Archived source code: http://doi.org/10.5281/zenodo.3236561 ^111^

License: MIT

### Phenotype-to-Genotype Mapping

Source code: https://github.com/NCBI-Hackathons/pheno_geno_ataxia

Archived source code: http://doi.org/10.5281/zenodo.3236569 ^112^

License: MIT

## Data availability

### Underlying data

All data underlying the results are available as part of the article and no additional source data are required.
