# Supplementary material for: Iron Hack - A symposium/hackathon focused on porphyrias, Friedreich’s ataxia, and other rare iron-related diseases
Source: F1000Res. 2019 Jul 19;8:1135. [Version 1] doi: 10.12688/f1000research.19140.1 (PMC6894363; doi:10.12688/f1000research.19140.1)
Supplement: Peer review history

# Peer review: Reviewer response for version 1

**DOI:** 10.5256/f1000research.20975.r56584

**Published:** 2019-12-04

**Authors:** Robert J. Desnick

**Affiliations:** 1 Department of Genetics and Genomic Sciences, Icahn School of Medicine at Mount Sinai, New York, NY, USA

**General Comments:**

 This is an interesting and insightful manuscript that describes several novel computer-based algorithms that can investigate and/or uncover new insights into the porphyrias and Fredrich Afaxia. It proves the usefulness of bringing together experts in various fields as a team to stove certain scientific problems. The Iron Hack was very successful and in a short time accomplished an amazing amount of work.

**Specific Comments:**

 Maturation and implementation of the various projects may prove the significance of their newly construction programs. Relevant to the porphyrias, it is important that UPWARD does not mislead symptomatic individuals to use consumer/commercial chips (e.g., 23andMe; Ancestry) to establish a diagnosis. This approach has already misguided people to believe they have a porphyria; However, they do not have the “gold standard” biochemical evidence of a specific porphyria. In fact the chip SNP data can lead to individuals having more than one porphyria based on being heterozygous in SNPs for two or more heme biosynthetic genes that are common and benign.

I confirm that I have read this submission and believe that I have an appropriate level of expertise to confirm that it is of an acceptable scientific standard.

# Peer review: Reviewer response for version 1

**DOI:** 10.5256/f1000research.20975.r51424

**Published:** 2019-08-28

**Authors:** Karl E. Anderson

**Affiliations:** 1 Department of Preventive Medicine and Community Health, The University of Texas Medical Branch at Galveston, Galveston, TX, USA

“Iron Hack” is a rare disease-focused hackathon. As a collaborative, problem-solving effort it has successfully attracted participants of diverse backgrounds at the U of South Florida and focusses on rare iron-related genetic diseases, specifically porphyrias and Friedreich’s ataxia. 

  

1. It is pitched at a general audience, but some of the material is probably too technical for a general audience. So any revisions should aim to further simplify and explain areas of discussion that are complex and technical.
2. Introduction: Friedreich’s ataxia – It would be useful to add a paragraph on diagnosis, and the underlying genetic cause.
3. The proposed projects are interesting and timely. These are (1) exploration of consumer-genomics data, (2) largescale RNAseq data mining, (3) genomic data visualization, (4) rare-disease variants discovery, and (5) genotype-to-phenotype mapping. These all seem innovative. The scope of work is very large, and it might be asked whether engaging a larger working group from many institutions would lead to more rapid progress. 

I confirm that I have read this submission and believe that I have an appropriate level of expertise to confirm that it is of an acceptable scientific standard.
